# Design and *Escherichia coli* Expression of a Natively Folded Multi-Disulfide Bonded Influenza H1N1-PR8 Receptor-Binding Domain (RBD)

**DOI:** 10.3390/ijms25073943

**Published:** 2024-04-01

**Authors:** Thao Tu, Tharangani Rathnayaka, Toshiyo Kato, Kenji Mizutani, Tomonori Saotome, Keiichi Noguchi, Shun-ichi Kidokoro, Yutaka Kuroda

**Affiliations:** 1 Department of Biotechnology and Life Science, Faculty of Engineering, Tokyo University of Agriculture and Technology, 2-24-16 Nakamachi, Koganei-shi 184-8588, Tokyo, Japan; s224725u@st.go.tuat.ac.jp (T.T.); tharangi15@yahoo.co.jp (T.R.); 2 NMR Group, Smart-Core-Facility Promotion Organization, Tokyo University of Agriculture and Technology, 2-24-16 Nakamachi, Koganei-shi 184-8588, Tokyo, Japan; fq4611@go.tuat.ac.jp (T.K.); knoguchi@cc.tuat.ac.jp (K.N.); 3 Graduate School of Medical Life Science, Yokohama City University, 1-7-29 Suehiro, Yokohama 230-0045, Kanagawa, Japan; mizutani@yokohama-cu.ac.jp; 4 Department of Materials Science and Bioengineering, Nagaoka University of Technology, 1603-1 Kamitomioka-cho, Nagaoka-shi 940-2188, Niigata, Japan; t_saotome@vos.nagaokaut.ac.jp (T.S.); kidokoro@vos.nagaokaut.ac.jp (S.-i.K.)

**Keywords:** influenza A, H1N1, receptor-binding domain, refolding, NMR, *E. coli* expression system, disulfide bonds

## Abstract

Refolding multi-disulfide bonded proteins expressed in *E. coli* into their native structure is challenging. Nevertheless, because of its cost-effectiveness, handiness, and versatility, the *E. coli* expression of viral envelope proteins, such as the RBD (Receptor-Binding Domain) of the influenza Hemagglutinin protein, could significantly advance research on viral infections. Here, we show that H1N1-PR8-RBD (27 kDa, containing four cysteines forming two disulfide bonds) expressed in *E. coli* and was purified with nickel affinity chromatography, and reversed-phase HPLC was successfully refolded into its native structure, as assessed with several biophysical and biochemical techniques. Analytical ultracentrifugation indicated that H1N1-PR8-RBD was monomeric with a hydrodynamic radius of 2.5 nm. Thermal denaturation, monitored with DSC and CD at a wavelength of 222 nm, was cooperative with a midpoint temperature around 55 °C, strongly indicating a natively folded protein. In addition, the ^15^N-HSQC NMR spectrum exhibited several ^1^H-^15^N resonances indicative of a beta-sheeted protein. Our results indicate that a significant amount (40 mg/L) of pure and native H1N1-PR8-RBD can be produced using an *E. coli* expression system with our refolding procedure, offering potential insights into the molecular characterization of influenza virus infection.

## 1. Introduction

*Escherichia coli* is the most extensively used expression system to produce recombinant protein, as it is simple, has a rapid growth rate, and a low cost of production [1]. However, *E. coli* lacks subcellular compartments essential for protein post-translational modifications like glycosylation and disulfide bridge formation, which are essential for the correct folding of a protein. The reducing environment of the *E. coli* cytoplasm is also not ideal for the formation of stable disulfide bonds in a protein. Thus, recombinant proteins expressed in *E. coli* are often misfolded [2,3,4,5]. Hence, generating a properly folded protein with two disulfide bonds poses a challenge due to the existence of three possible disulfide connectivity patterns arising from four cysteine residues, with only one of them being the native configuration. In this research, we chose the two-disulfide-bonded receptor-binding domain (RBD) of Influenza A virus (A/Puerto Rico/8/1934(H1N1) as it is an important protein fragment for influenza studies.

According to the World Health Organization (WHO), the influenza virus is the cause of annual flu epidemics in humans, which cause around 5 million severe illnesses and 300,000–500,000 deaths each year [6]. Humans infected with the influenza virus suffer from a wide range of symptoms, from minor respiratory infections to potentially fatal pneumonia brought on by the virus or secondary bacterial infections affecting the lower respiratory tract [7].

Vaccination is the best prevention against influenza infection [8]. A promising alternative to egg-based vaccines [9] are recombinant subunit (protein) vaccines produced in various expression systems [10]. As for influenza, hemagglutinin (HA), which is exposed on the surface of the influenza virus, is the antigen used in most subunit vaccines [11]. Here, we selected the receptor-binding domain (RBD) of the HA protein as it is essential for the viral infection [12], and, based on previous findings, we predicted that its structure would remain natively folded upon protein dissection [13,14] and preserve its biophysical properties [15]. Indeed, RBD plays a vital role in binding the influenza virus to receptors on the host cell’s surface, with a highly conserved sequence containing several antigenic sites, thus holding great promises as an antigen for a recombinant subunit vaccine.

In this study, we developed a protocol to produce natively folded RBD of the influenza A H1N1 PR8 virus protein in the *E. coli* expression system based on the pET-15b expression vector. The final yield was 40 mg of H1N1-PR8-RBD’s lyophilized protein from 1 L of LB culture. H1N1-PR8-RBD’s biophysical and biochemical properties were analyzed with circular dichroism, fluorescence, light scattering, analytical ultracentrifugation, differential scanning calorimetry and NMR spectroscopy. The results strongly suggested that H1N1-PR8-RBD dissected from HA is natively folded and, thus, could be used to enhance our understanding of influenza virus infection at a molecular level.

## 2. Results

### 2.1. Expression, Refolding, and Purification

**Figure 1:**
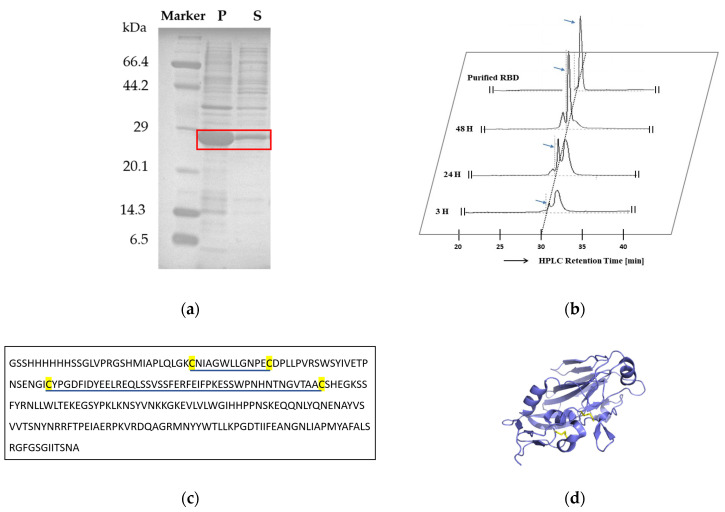
Expression and purification of H1N1-PR8-RBD. (**a**) SDS-PAGE of large-scale expression analysis. P: pellet fraction and S: soluble fraction. Red square indicates the H1N1-PR8-RBD bands. (**b**) RP-HPLC elution profiles of the protein purified after air-oxidation after 3 h, 24 h, and 48 h at 25 °C and after lyophilization. Blue arrows indicate the correctly folded H1N1-PR8-RBD peak. (**c**) Amino acid sequence of H1N1-PR8-RBD with 4 cysteine residues highlighted in yellow. Blue lines indicate disulfide-bonded cysteine residues. (**d**) Ribbon model of H1N1-PR8-RBD generated with PyMOL [18] with the coordinate of the PDB ID 1RU7. Disulfide bonds are presented in yellow lines (Cys59–Cys71 and Cys94–Cys139).

The recombinant H1N1-PR8-RBD protein, which has 243 amino acid residues and two disulfide bonds (Cys59–Cys71 and Cys94–Cys139 from PDB ID 1RU7 [16]), was expressed in *E. coli* (Figure 1c,d). The yield of H1N1-PR8-RBD after dialysis was high (70–100 mg/L), and SDS-PAGE analysis showed that H1N1-PR8-RBD was mainly expressed in the pellet fraction (Figure 1a).

After ultrasonication, the expressed protein pellet was oxidized in 6 M GuHCl in 50 mM Tris–HCl by stirring with a magnetic stirrer at 25 °C in 3 h and was then purified with Ni-NTA chromatography and RP-HPLC. RP-HPLC analysis showed that the SS bonds of H1N1-PR8-RBD are barely formed upon expression (Figure 1b). Hence, the incubation time was extended to 24 h and 48 h to ensure complete oxidation. After 24 h, the analytical RP-HPLC elution profile presented two peaks, which indicated the presence of mixed disulfide bond patterns. However, after 48 h, the broad peak completely disappeared, leaving one major sharp peak (Figure 1b). This main peak was collected with large-scale RP-HPLC and lyophilized. Analytical RP-HPLC after lyophilization showed a sharp single peak (Figure 1b), suggesting the presence of a single disulfide-bound species [17]. The peak eluted approximately one minute before the fully reduced H1N1-PR8-RBD (Figure S2). Thus, our expression and refolding procedure produced a single RP-HPLC peak with correct folding and structure, as demonstrated with the biophysical characterization below.

### 2.2. Conformational Characterizations

**Figure 2:**
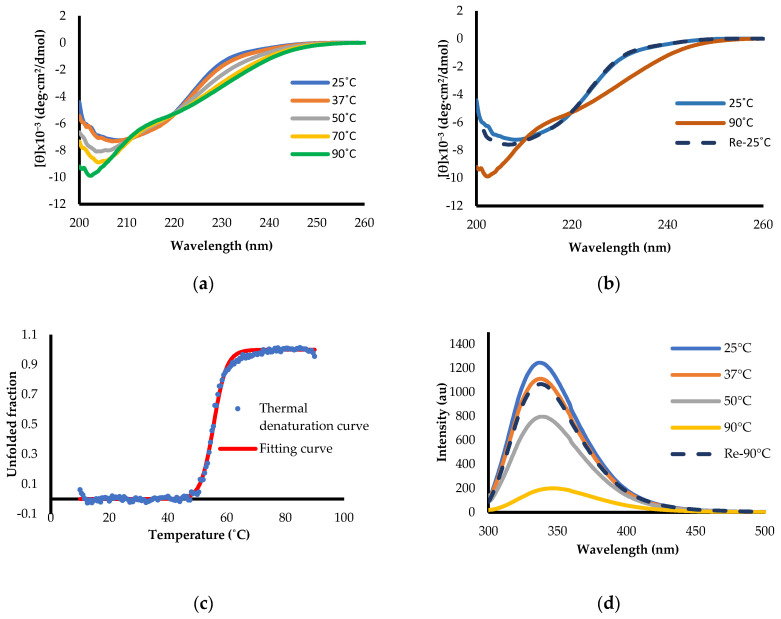
Biophysical properties of H1N1-PR8-RBD. (**a**) The secondary structures of RBD were measured with far-UV CD spectroscopy (200–260 nm) from 25 °C to 90°C with 20 °C increments. (**b**) The reversibility was assessed with CD spectra measured by first measuring the spectrum at 25 °C, 90 °C, and after cooling back to 25 °C. (**c**) The thermal denaturation of H1N1-PR8-RBD monitored with CD at 222 nm. Blue dots represent raw data. (**d**) Tryptophan fluorescence intensity of H1N1-PR8-RBD measured from 25 °C to 90 °C.

Far-UV circular dichroism indicated that 10.6% and 40.3% of the residues formed α-helix and β-sheet, respectively, which was close to the secondary structure content calculated from the crystal structure (PDB-ID: 1RU7; Figure S3). CD measured at temperatures from 25 °C to 90 °C indicated a reversible thermal denaturation (Figure 2a,b). The thermal denaturation curve monitored with CD at a wavelength of 222 nm was S-shaped, indicating a cooperative unfolding, as expected for a natively folded globular protein, with a midpoint temperature of 55.5 °C (Figure 2c). Next, the tryptophan fluorescence intensity of H1N1-PR8-RBD at 25 °C showed an emission maximum at 337 nm and shifted to 347 nm at 90 °C, indicating a 10 nm redshift with an 84% intensity decrease (Figure 2d), which is a typical change upon protein unfolding [19].

**Figure 3:**
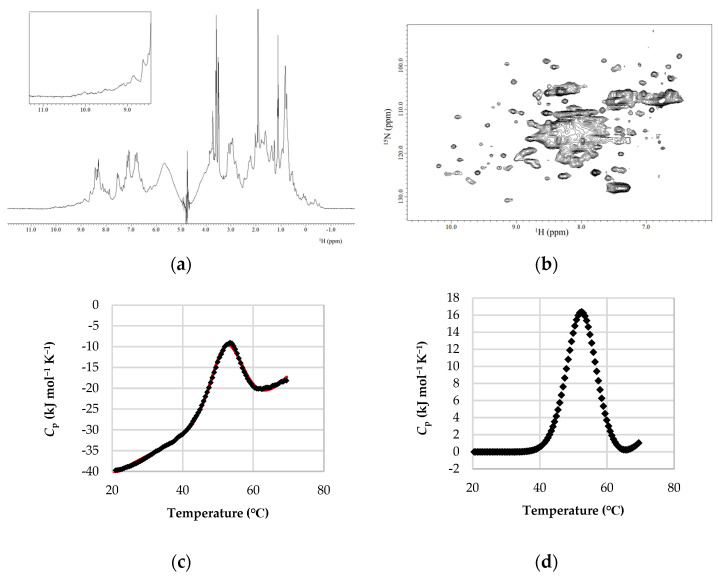
NMR spectroscopy and DSC thermogram of H1N1-PR8-RBD. (**a**) One-dimensional (1D) and (**b**) two−dimensional (2D) NMR spectroscopy measurements of H1N1-PR8-RBD were conducted at 20 °C in sodium acetate buffer with pH 4.7. (**c**) DSC thermogram of H1N1-PR8-RBD in 10 mM sodium acetate buffer (pH 4.7). The protein concentration was 0.80 mg/mL, and the scan rate was +1.0 °C/min. The black dots and red line represented the raw data and fitting curve of DSC thermogram, respectively. (**d**) The baseline correction of DSC thermogram by assuming that the heat capacity at native state was zero.

The ^1^H-NMR spectrum showed scattered, sharp peaks typical of a natively folded structure. Amide and methyl protons with, respectively, chemical shifts of 7.0–9.0 ppm and below 0 ppm were observed, strongly supporting a well-folded tertiary structure (Figure 3a). In addition, the ^15^N-HSQC spectrum measured at 20 °C also showed well-dispersed and sharp cross-peaks, again suggesting a natively folded structure (Figure 3b). In DSC measurements, a sharp, single endothermic peak was observed in the thermogram (Figure 3c). It indicated that the protein was natively folded and cooperatively unfolded via heating. DDCL3 analysis of DSC thermograms calculated that the midpoint temperature (*T*_m_) was 54.72 °C and the calorimetric enthalpy (Δ*H*(*T*_m_)) was 258.51 kJ/mol. The *T*_m_ value of the DSC measurements coincided with the CD measurements (Figure 2c), and it implied that its thermal denaturation occurred with a two-state model between the native state and denatured state (Figure 3d).

### 2.3. Particle Size and Solubility

**Figure 4:**
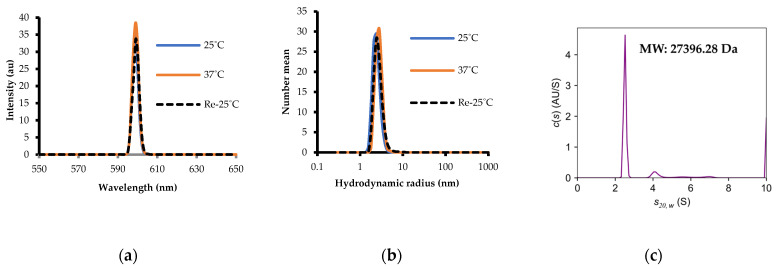
Solubility and particle size of H1N1-PR8-RBD and its oligomers. (**a**) Static light scattering. (**b**) Dynamic light scattering. Note that the SLS and DLS spectra at 25 °C (blue line) and Re-25° (broken line) fully overlapped. (**c**) Sedimentation kinetics analysis via analytical ultracentrifugation (AUC).

The solubility of the purified H1N1-PR8-RBD was assessed with SLS, and the particle size of the aggregates was measured with DLS and AUC. H1N1-PR8-RBD had low SLS intensities at 25 °C, suggesting no aggregation. The SLS intensity increased slightly when the temperature was increased to 37 °C and returned to the initial spectrum when the temperature decreased to 25 °C, indicating a good reversibility (Figure 4a). DLS measurements indicated a protein hydrodynamic radius [*R*_h_] of around 2.5 nm at 25 °C, which is appropriate for a protein of this size in a monomeric state [20] (Figure 4b). Analytical ultracentrifugation data also indicated that 99.361% of the protein was monomeric with a molecular weight (MW) approximately equal to the theoretical MW of 27.44 kDa calculated with the ProtParam tool (Figure 4c) [21].

### 2.4. Biochemical Stability

**Figure 5:**
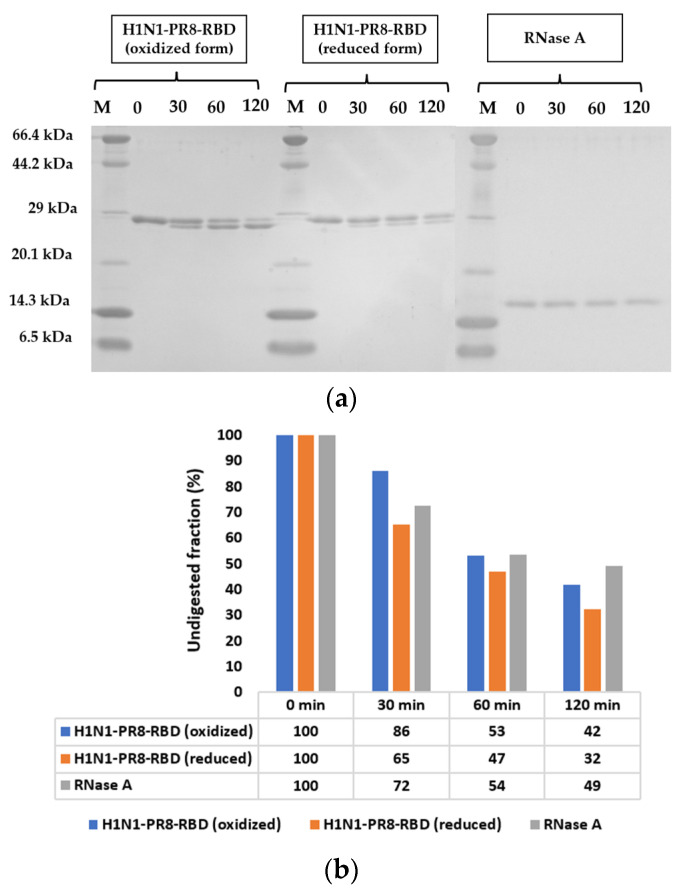
Biochemical stability of H1N1-PR8-RBD assessed with limited proteolysis. (**a**) SDS-PAGE results of H1N1-PR8-RBD and RNase A. M represents the marker. (**b**) Band intensities before and after trypsin digestion. The undigested fraction was calculated with CS Analyzer 4 software v4.0, based on SDS-PAGE image.

Protease digestion is widely used to evaluate the biochemical stability of proteins [22,23,24]. We evaluated the protein stability by assessing its resistance against trypsin digestion. RNase A protein with high stability and a well-folded structure was used as a positive control while the reduced form of H1N1-PR8-RBD was used as a negative control. The oxidized H1N1-PR8-RBD was not completely digested by trypsin after 120 min incubation, with an undigested fraction of 42%, which was 7% less than the undigested fraction of the well-folded RNase A (49%; Figure 5b). According to the calculation of trypsin cleavage sites on the H1N1-PR8-RBD protein via PeptideCutter (https://web.expasy.org/peptide_cutter/ (accessed on 23 December 2023)) (Table S1), trypsin may cut the protein at either (1) the N-terminus and remove the Histidine tag (GSSHHHHHHSSGLVPR), (2) at the C-terminus and remove the GFGSGIITSNA fragment, or (3) at both sites mentioned above. This resulted in a lower-size band with minor difference in the molecular weight and almost the same structure as the higher-size band. The reduced form of H1N1-PR8-RBD, on the other hand, was digested differently, which could possibly stem from the cleavage of the disulfide bonds that unfolded the protein and led to the exposition of the peptide back bone, making it more accessible to trypsin cleavage [22,25].

## 3. Discussion

*Escherichia coli* is an excellent system for expressing large amounts of recombinant proteins in a short timeframe due to its high yield and rapid growth [3]. We considered utilizing this platform to produce H1N1-PR8-RBD, foreseeing potential challenges in protein folding within the reducing environment of *E. coli*, particularly considering the presence of two disulfide bonds in H1N1-PR8-RBD. Hence, in our study, RP-HPLC is not only a method of choice for obtaining protein with high purity, but also for monitoring the refolding of SS-bound proteins [26]. Efficient refolding of SS-bonded proteins is a competition between folding and aggregation resulting from incorrectly formed SS bonds. Therefore, the refolding conditions must be optimized for each protein, considering its ability to fold into the native state, the rate of SS bond formation (which is essentially irreversible in an air-oxidization protocol, unless there are non-SS-bonded cysteines with free sulfhydryl), and the solubility of the protein [27]. In our research, RP-HPLC analysis revealed an increase in the formation of correct disulfide bonds after prolonged air oxidation in GuHCl. GuHCl increases a protein’s solubility but favors the unfolded state. Thus, air oxidation in GuHCl usually results in multiple RP-HPLC peaks corresponding to the formation of non-native SS bonds, underscoring the difficulty of achieving properly folded protein in *E. coli* [28]. Thus, the appearance of a single RP-HPLC peak after oxidization in GuHCl was surprising (Figure S2). We rationalized this observation with the fact that the SS-bonds form between the nearest cysteines. A rough estimate indicates that Cys59–Cys71 (native pair) is eight times more likely to form than Cys71–Cys94 (non-native) since the inter-residue distances are, respectively, 12 and 23 (which represent a factor of 2) and assuming that the chain is fully flexible. Eventually, thanks to the favorable intercysteine distances, together with our optimization, the elution profile of H1N1-PR8-RBD after RP-HPLC purification showed a single sharp peak, representing a single disulfide bond pattern, which was confirmed as the native disulfide bond pairing of the protein [29].

The protein’s native structure was confirmed through conformational characterizations employing various spectroscopic measurements. The secondary structure contents of the *E. coli*-expressed H1N1-PR8-RBD assessed with CD were highly similar to the contents calculated from the crystal structure of the hemagglutinin protein. The thermal denaturation assessed with CD at 222 nm showed an S-shape, with a cooperative transition suggesting the well-folded structure of the protein. Tryptophan fluorescence data showed a red shift as the temperature increased to 90 °C, indicating the protein’s unfolding at high temperature, which is a typical characteristic of a native protein [30]. In addition, the proton NMR and ^15^N-HSQC NMR data both indicated that the recombinant H1N1-PR8-RBD has a native tertiary structure. The DSC measurements revealed a single and sharp endothermic peak, again confirming the protein’s native folding and indicating that its thermal denaturation followed a two-state model.

All the performed particle size analyses, including DLS, SLS, and AUC, indicated that the protein is monomeric. Specifically, DLS and AUC measurements revealed a hydrodynamic radius of approximately 2.5 nm for H1N1-PR8-RBD, while SLS intensity showed no evidence of aggregations.

In addition, we also used trypsin digestion to assess the biochemical stability of H1N1-PR8-RBD with RNase A as a positive control, because of its well-folded structure and high stability, and the reduced H1N1-PR8-RBD as a negative control. The oxidized form of H1N1-PR8-RBD showed an approximate resistance against trypsin digestion via RNase A, and the reduced form was digested into smaller fragments compared to the oxidized form, which was probably due to the exposure of proteolytic cleavage sites resulting from the cleavage of disulfide bonds. This suggested that the stability of the oxidized H1N1-PR8-RBD against trypsin digestion possibly stems from the natively folded structure of the protein.

It is noteworthy that, in previous studies, the majority of recombinant hemagglutinin proteins were expressed using insect cell systems (such as the Baculovirus Expression Vector System—BEVS), mammalian cell systems (e.g., CHO and HEK293 cells), or plant-based expression systems [13,31,32,33,34]. Only a limited number of studies reported on the bacterial expression systems (specifically *E. coli*) production of HA protein due to difficulties in the folding process. However, even when the *E. coli* system was employed, only one study has documented the expression of the RBD dissected from the full-length HA protein without utilizing fusion proteins. This reported RBD (PDB ID 3MLH) originated from the H1N1-pdm09 strain [12], distinct from our chosen H1N1-PR8 strain that is renowned as one of the most utilized strains in vaccine and therapeutic research. Our study stands out as one of the few studies to successfully produce an isolated influenza receptor-binding domain in *E. coli* with 100% purity and a significant yield of lyophilized protein. The recombinant protein’s native structure and folding have been confirmed through various techniques.

## 4. Materials and Methods

### 4.1. Plasmid Construction, Protein Expression, Refolding, and Purification

A DNA sequence encoding H1N1-PR8-RBD was inserted between the NdeI and XhoI regions on a pET15b plasmid vector, with a 6xHistidine-tag designed at the N-terminal (Figure S3a). *E. coli* DH5α was used for cloning. The protein was expressed in *E. coli* BL21(DE3) cells with a Luria–Bertani medium. The cells were cultured at 37 °C with 250 rpm shaking. Protein expression was induced via the addition of 1.0 mM isopropyl β-D-1-thiogalactopyranoside (IPTG) when the OD at 590 nm reached 0.5–0.6 [26]. After 6 h, the cells were harvested via centrifugation (8000 rpm, 20 min, and 4 °C) with a Hitachi himac CF16RX centrifuge (Hitachi, Tokyo, Japan) and a T9A31 angle rotor. Following that, the cells were lysed in a lysis buffer (50 mM Tris-HCl pH 8.0 and 150 mM NaCl) and lysis wash buffer (2 M Tris-HCl pH 8.0, 1% NP-40 (*v*/*v*), 0.1% deoxycholic acid (*w*/*v*), and 0.5 M EDTA) via ultrasonication. The expressed protein in the inclusion bodies was collected via centrifugation (8000 rpm, 20 min, and 4 °C). The pellet was stirred with a magnetic stirrer in a tube containing 6 M GuHCl in 50 mM Tris–HCl, with a pH of 8.8 at 25 °C. The dissolved protein was centrifuged (8000 rpm, 20 min, and 4 °C) and filtered through a 0.22 µm filter (Millex-GV; Millipore, Burlington, MA, USA) to remove any aggregates. The samples were purified using an open Ni-NTA column (Wako, Tokyo, Japan). The column was washed three times with the wash buffer (6 M GuHCl, 10 mM Tris-HCl, and pH 6.8), and H1N1-PR8-RBD was eluted with the elution buffer (6 M GuHCl and 10% acetic acid). The eluted protein sample was dialyzed against reverse osmosis (RO) water using a dialysis membrane with a molecular weight cut-off (MWCO) of 14 kDa to remove GuHCl. Dialysis was performed at 4 °C overnight, with the outer solution changed 3 times, at 30 min, 60 min, and 2 h after starting dialysis. The protein concentration was measured at 280 nm using Nanodrop (Thermo Fisher Scientific, Waltham, MA, USA). Large-scale RP-HPLC was used to purify H1N1-RBD. Protein purities were confirmed with analytical reversed-phase HPLC. The protein was stored as a lyophilized powder at −30 °C for later use. The molecular weight of the protein was confirmed with MALDI-TOF mass spectroscopy on an Autoflex speed TOF/TOF instrument (Bruker Daltonics, Billerica, MA, USA) (Figure S1).

### 4.2. Analytical Reverse-Phase High-Performance Liquid Chromatography (RP-HPLC)

The proteins were analyzed with reverse-phase (RP) high-performance liquid chromatography (HPLC; Shimadzu, Kyoto, Japan) using an Intrada 5WP-RP column (Imtakt, Kyoto, Japan). Solution A (MilliQ-water + 0.1% trifluoroacetic acid (TFA)) and Solution B (Acetonitrile + 0.05% TFA) served as a mobile phase, with a flow rate of 1 mL/min. The column temperature used was 30 °C. The protein powder was dissolved at a concentration of 0.2 mg/mL with acetic acid at a final concentration of 10% (*v*/*v*) and filtered with a 0.20 µm membrane filter (MDI Membrane Technology, Ambala Cantt, India) to remove any aggregates. The reduced form of H1N1-PR8-RBD was prepared at the same protein concentration, with the addition of 1 M DTT, and incubated at 37 °C for one hour.

### 4.3. Circular Dichroism (CD) Measurements

Samples were prepared by dissolving the lyophilized protein in MQ, and the protein concentration of the samples were adjusted to 0.2 mg/mL in 10 mM MES buffer with a pH of 6.0. The secondary structure of H1N1-RBD was measured using a 2 mm path-length quartz cuvette (TOSOH, Tokyo, Japan) in a continuous scanning mode with a JASCO-J820 CD spectropolarimeter (JASCO, Tokyo, Japan). For each measurement, three scans were accumulated from 200 to 260 nm wavelength at temperatures of 25 °C, 37 °C, 50 °C, and 90 °C. The reversibility of H1N1-PR8-RBD was assessed by measuring the spectrum after cooling the sample to 25 °C. The secondary structure content was calculated using BeStSel [35]. The protein thermal stability was also assessed at the same pH and protein concentration. The scan rate was 1 °C/min and monitored from 10 to 90 °C at a wavelength of 222 nm. Midpoint temperature was computed with the least-square fitting of the experimental data, assuming a two-state model using Origin 2023 (OriginLab Corp, Northampton, MA, USA) [36].

### 4.4. Light Scattering Measurements

The stock solutions were prepared by dissolving the lyophilized protein in MilliQ. After that, the stock solutions were centrifuged at 20,000× *g* for 20 min at 4 °C and filtered through a 0.20 µm filter to remove any aggregates. The final samples were prepared by diluting the stock solutions to 0.3 mg/mL in 10 mM MES buffer with a pH of 6.0. The dynamic light scattering (DLS) measurements were performed at 25 °C and 37 °C and then reversed to 25 °C, using a polystyrene cuvette with a Zeta-nanosizer (Malvern Panalytical, Malvern, UK). The hydrodynamic radius [*R*_h_] was determined from the size distribution using the Stokes–Einstein equation and was averaged over three individual measurements. Static light scattering (SLS) measurements were performed under the same conditions at an excitation wavelength of 600 nm with an FP-8500 spectrofluorometer (JASCO, Tokyo, Japan) using a quartz microcuvette with an optical path length of 3 mm (TOSOH, Tokyo, Japan) [37].

### 4.5. Fluorescence Spectroscopy Measurements

Samples were prepared by dissolving lyophilized proteins in MQ. After that, samples were centrifuged at 20,000× *g* for 20 min at 4 °C and filtered through a 0.20 µm filter to remove any aggregates. The protein concentrations of the samples were adjusted to 0.3 mg/mL in 10 mM MES with a pH of 6.0. The tryptophan fluorescence spectra with an excitation wavelength of 295 nm were measured using a quartz cuvette with a 3 mm optical path length (TOSOH, Tokyo, Japan) and an FP-8500 spectrofluorometer (JASCO, Tokyo, Japan). The temperature range was increased from 25 °C to 90 °C and finally decreased back to 25 °C to assess reversibility.

### 4.6. Nuclear Magnetic Resonance (NMR) Spectroscopy

One-dimensional (1D) and two-dimensional (2D) NMR spectroscopy measurements of H1N1-PR8-RBD were conducted at 20 °C in a sodium acetate buffer with a pH of 4.7. For 1D measurements (^1^H NMR), the lyophilized nonlabelled protein powder was dissolved to a concentration of 0.3 mM in a 10 mM sodium acetate buffer with a pH of 4.7 and 10% (*v*/*v*) D_2_O. For 2D measurements (heteronuclear single-quantum correlation spectroscopy—HSQC), ^15^N-enriched H1N1-PR8-RBD protein powder was dissolved to a concentration of 0.2 mM in a 10 mM deuterated sodium acetate buffer (Sigma-Aldrich, Burlington, MA, USA) with a pH of 4.7 and 10% (*v*/*v*) D_2_O. The samples were then transferred to a 5 mm Shigemi microtube (Shigemi Co., Ltd., Tokyo, Japan). NMR spectra were acquired on a 500 MHz spectrometer (JEOL JNM-ECA500) (JEOL, Tokyo, Japan) through 512 scans for 1D- and 256 scans for 2D-measurements. The 1D NMR spectra were acquired with spectral widths of 12.2 ppm to −2.8 ppm; the 2D NMR spectra were acquired with spectral widths of 12.2 ppm to −2.8 ppm in *f_2_* (^1^H) and 140–90 ppm in *f*_1_ (^15^N). A total of 1024 complex points were collected in the ^1^H dimension and 128 complex points in the ^15^N dimension for 2D measurement. WATERGATE was performed beforehand for both 1D- and 2D-measurements to suppress water signals.

### 4.7. Analytical Ultracentrifugation (AUC) Measurements

Lyophilized protein powder was dissolved in MilliQ water (MQ) to achieve a protein concentration of 1.5 mg/mL. The samples were dialyzed using a Spectra/Por 3 membrane with a molecular weight cutoff (MWCO) of 14 kDa for 18 h at 4 °C in 10 mM MES buffer (pH 6.0). Two buffer changes were performed during the dialysis process. After dialysis, the protein concentration was adjusted to 1 mg/mL using the dialysis buffer. In order to eliminate aggregates, the samples were filtered through 0.22 μm membrane filters (Millex-GV; Millipore, Burlington, MA, USA).

Prior to conducting the experiments, protein concentrations and pH values of the samples were confirmed. Sedimentation velocity experiments were carried out at 20 °C using an Optima XL-I analytical ultracentrifuge (Beckman-Coulter; Indianapolis, IN, USA) equipped with an eight-hole An-50 Ti rotor. The samples were centrifuged at a rotor speed of 50,000 rpm. The sedimentation velocity data were analyzed using the continuous sedimentation coefficient distribution model c(S) analysis module in the SEDFIT software v16.1c [38]. The c(S) distribution was then converted into c(M), representing a molar mass distribution. Solvent density, viscosity, and protein partial specific volumes were calculated with SEDNTERP software v3.0 [39].

### 4.8. Limited Proteolysis

The biochemical stability of H1N1-PR8-RBD was assessed with limited proteolysis. A concentration of 0.127 mg/mL (~4.7 µM) of the oxidized and reduced form of H1N1-PR8-RBD was digested in 200 ng/mL of trypsin in 10 mM of MES buffer at a pH of 6.0. The oxidized form was prepared by dissolving the lyophilized protein powder in MQ then centrifuging at 20,000× *g* at 4 °C for 20 min and filtered with a 0.20 µm filter. The reduced form was prepared by incubating the protein solution with 1 M dithiothreitol (DTT) for 90 min. After that, the sample was dialyzed against 1 L of 2 mM DTT for 16 h (changing the buffer two times) to remove DTT but still retain the reduced form of the protein. The digestion mixtures were incubated for 30, 60, and 120 min at 37 °C. At the end of each incubation period, a 20 µL aliquot of the mixtures was mixed with loading buffer containing β-mercaptoethanol and heated at 95 °C for 5 min to stop the reaction. The proteolysis was examined with SDS-PAGE and band intensities were assessed with ATTO Image Analysis Software (CS Analyzer 4) (ATTO, Tokyo, Japan). RNase A from Bovine pancreas at a concentration of 0.065 mg/mL (~4.7 µM) was prepared the same way as the H1N1-PR8-RBD and used for comparison of the stability against trypsin digestion [40].

### 4.9. Differential Scanning Calorimetry (DSC)

A total of 2 mg of lyophilized protein powder was dissolved in 2 mL of MQ and dialyzed in 1 L of 10 mM Na-Acetate buffer (pH 4.7) at 4 °C for 15 h. The outer solution was changed only once, 3 h after the start of dialysis. The dialyzed sample was filtered by using a 0.20-μm sterilized filter (Sartorius, Göttingen, Germany) to remove aggregates, and the protein concentration was measured with UV-spectrophotometry. Finally, the sample was ultrasonically degassed for 3 min.

DSC measurements were performed using a VP-DSC microcalorimeter (MicroCal, MA, USA) at a scan rate of +1.0 °C/min in the temperature range of 20 to 80 °C. First, blank measurements were performed using the buffer, which is the outer solution of the final dialysis. Next, the thermal unfolding of the protein sample was assessed with DDCL3 analysis [41,42]. DDCL3 determined the midpoint temperature (*T*_m_) and the calorimetric enthalpy ((Δ*H*(*T*_m_)) by analyzing the apparent heat capacity curves using a nonlinear, least-square fitting algorithm with a two-state model [41,42].

## 5. Conclusions

The recombinant receptor-binding domain of H1N1-PR8 (strain A/Puerto Rico/8/1934 H1N1) was successfully expressed in *Escherichia coli* with high yield, and the purified protein exhibited a native structure and biophysical characteristics. This highlights the strategy to produce a multi-disulfide bonded protein in *E. coli*, which not only utilizes the advantages of this expression system but also results in a recombinant protein with native properties. We believe that our natively folded H1N1-PR8-RBD will be useful for developing subunit vaccine antigens in the near future.

## Data Availability

The data generated and analyzed during the current study are available from the corresponding author on reasonable request.

## Supplementary Materials

The following supporting information can be downloaded at: https://www.mdpi.com/article/10.3390/ijms25073943/s1.
