# Supplementary material for: Design and Escherichia coli Expression of a Natively Folded Multi-Disulfide Bonded Influenza H1N1-PR8 Receptor-Binding Domain (RBD)
Source: Int J Mol Sci. 2024 Apr 1;25(7):3943. doi: 10.3390/ijms25073943 (PMC11012049; doi:10.3390/ijms25073943)
Supplement: Supplementary file 1 [file ijms-25-03943-s001.zip › ijms-28710680-supplementary.pdf]

Supplementary Materials

Figure S1. Plasmid construction and MALDI-TOF mass spectroscopy of H1N1-PR8-RBD.

Figure S2. RP-HPLC elution profiles of the oxidized and reduced H1N1-PR8-RBD.

Figure S3. The secondary structure content of H1N1-PR8-RBD.

Table S1. H1N1-PR8-RBD cleavage sites by trypsin calculated by PeptideCutter.

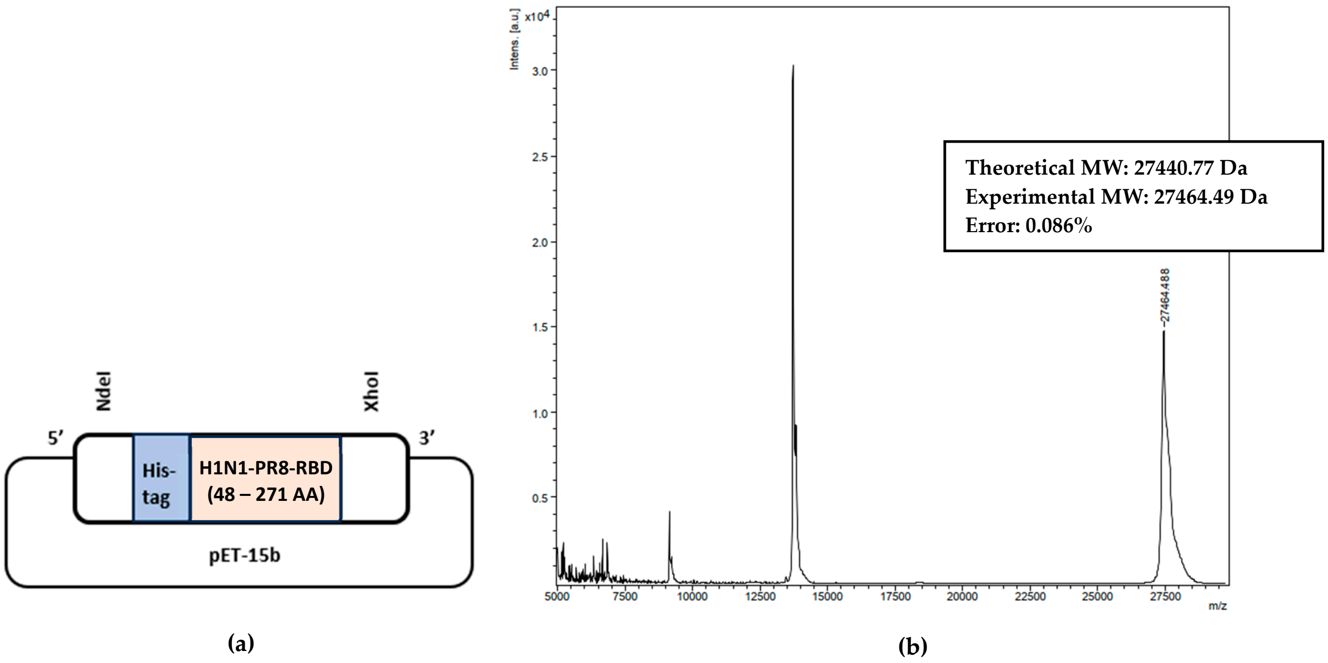

Figure S1. Plasmid construction and MALDI-TOF mass spectroscopy of H1N1-PR8-RBD. (**a**) pET-15b vector of H1N1-PR8-RBD; (**b**) MALDI-TOF mass spectroscopy of H1N1-PR8-RBD. The protein's molecular weight was determined using matrix-assisted laser desorption/ionization-time of flight (MALDI-TOF) mass spectrometry on an Autoflex speed TOF/TOF instrument (Bruker Daltonics, Billerica, MA, USA). To prepare the matrix solution, 10 mg of sinapic acid was dissolved in 1 mL of a solution containing 300 μL of acetonitrile, 100 μL of 1% trifluoroacetic acid, and 600 μL of Milli Q water. Protein samples were prepared by mixing 1 μL of protein solution with 9 μL of the matrix solution. Subsequently, a gradient of 10 μM, 1 μM, and 0.1 μM concentrations were spotted onto the MALDI-TOF MS plate and allowed to air-dry. The experiment molecular

weight was measured with MALDI-TOF mass spectroscopy, and the theoretical molecular weight was calculated using ProtParam (https://web.expasy.org/protparam/ (accessed on 04/03/2023)), relative error given.

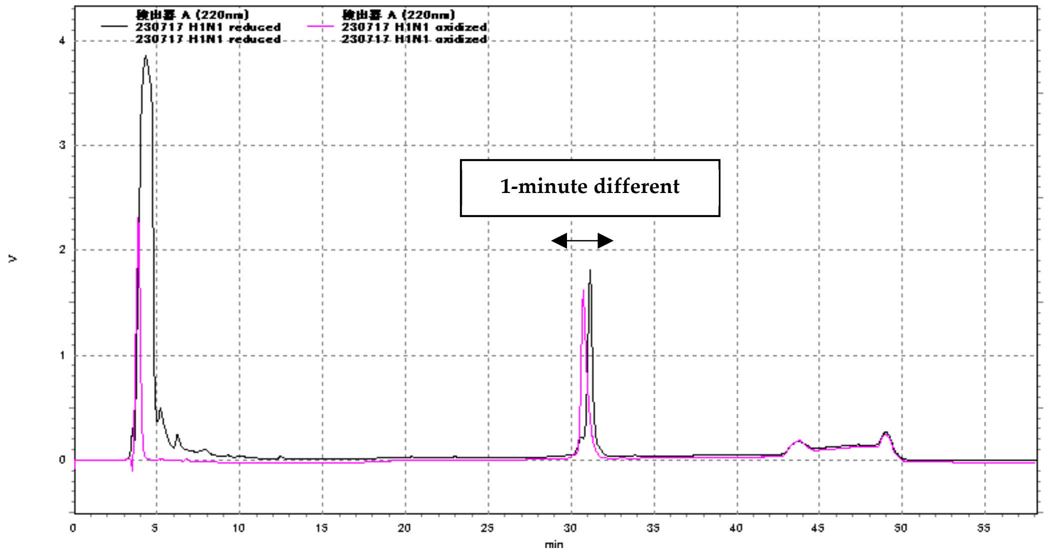

Figure S2. RP-HPLC elution profiles of the oxidized and reduced H1N1-PR8-RBD. The pink peak represents the oxidized RBD, while the black peak represents the reduced RBD obtained by incubating 1 hour with 1M DTT.

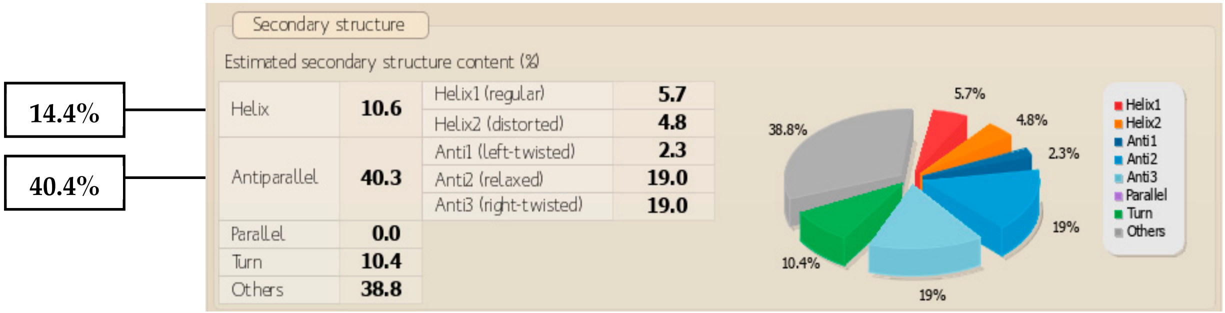

Figure S3. The secondary structure content of H1N1-PR8-RBD. The secondary structure content was calculated using BeStSel. The values in the box indicate the secondary structure content obtained from RBD region of hemagglutinin crystal structure PDB ID 1RU7.

Table S1. H1N1-PR8-RBD cleavage sites by trypsin calculated by PeptideCutter (https://web.expasy.org/peptide_cutter/) (accessed on 23/12/2023)). The calculation was performed based on H1N1-PR8-RBD amino acid sequence. Two accessible cleavage sites by trypsin at the N- and C-termini of the oxidized H1N1-PR8-RBD are highlighted.

Position of cleavage site	Name of cleaving enzyme(s)	Resulting peptide sequence	Peptide length [aa]	Peptide mass [Da]
16	Trypsin	GSSHHHHHHSSGLVPR	16	1768.875
28	Trypsin	GSHMIAPLQLGK	12	1251.509
48	Trypsin	CNIAGWLLGNPECDPLLPVR	20	2180.568
76	Trypsin	SWSYIVETPNSENGICYPGDFIDYEELR	28	3297.553
87	Trypsin	EQLSSVSSFER	11	1268.346
93	Trypsin	FEIFPK	6	779.934
114	Trypsin	ESSWPNHNTNGVTAACSHEGK	21	2226.321
119	Trypsin	SSFYR	5	658.712
127	Trypsin	NLLWLTEK	8	1016.205
133	Trypsin	EGSYPK	6	679.728
135	Trypsin	LK	2	259.349
141	Trypsin	NSYVNK	6	723.784
142	Trypsin	K	1	146.189
144	Trypsin	GK	2	203.241
159	Trypsin	EVLVLWGIHHPPNSK	15	1726.011
181	Trypsin	EQQNLYQNENAYVSVVTSNYNR	22	2633.770
182	Trypsin	R	1	174.203
192	Trypsin	FTPEIAERPK	10	1187.361
194	Trypsin	VR	2	273.335
199	Trypsin	DQAGR	5	545.553
232	Trypsin	MNYYWTLLKPGDTIIFEANGNLIAPMYAFALSR	33	3795.429
243	**end of sequence**	GFGSGIITSNA	11	1023.110
